# Review no. 2: a beginner’s guide for calculating eGFR slope using linear mixed-effects model in R—step-by-step methods and code examples

**DOI:** 10.1007/s10157-026-02835-8

**Published:** 2026-02-26

**Authors:** Megumi Oshima, Masahiko Gosho, Masao Iwagami, Yuka Sugawara, Hajime Nagasu, Takashige Kuwabara, Tadashi Sofue, Naoki Nakagawa, Yoshihisa Miyamoto

**Affiliations:** 1 Department of Nephrology and Rheumatology, Kanazawa University, Kanazawa, Ishikawa Japan; 2 Department of Biostatistics, Institute of Medicine, University of Tsukuba, Tsukuba, Ibaraki Japan; 3 Social Medicine Group, Department of Digital Health, Institute of Medicine, University of Tsukuba, Tsukuba, Ibaraki Japan; 4 Department of Non-Communicable Disease Epidemiology, Faculty of Epidemiology and Population Health, London School of Hygiene and Tropical Medicine, London, UK; 5 Division of Nephrology and Endocrinology, The University of Tokyo, Tokyo, Japan; 6 Department of Nephrology and Hypertension, Kawasaki Medical School, Kurashiki, Okayama Japan; 7 Department of Nephrology, Kumamoto University Graduate School of Medical Sciences, Kumamoto, Japan; 8 Department of Cardiorenal and Cerebrovascular Medicine, Faculty of Medicine, Kagawa University, Takamatsu, Japan; 9 Division of Cardiology and Nephrology, Department of Internal Medicine, Asahikawa Medical University, Asahikawa, Japan; 10 Department of Real-World Evidence, The University of Tokyo, Tokyo, Japan

**Keywords:** Real-world data, Chronic kidney disease, eGFR slope, Linear mixed-effects model

## Abstract

Estimated glomerular filtration rate (eGFR) slope, which quantifies the annual change in kidney function using longitudinal measurements of eGFR, has emerged as a critical surrogate endpoint for chronic kidney disease progression in clinical trials. Accordingly, there is an increasing number of observational studies calculating the eGFR slope as an outcome or exposure of interest. To calculate the eGFR slope, linear mixed-effects models are considered statistically more efficient and therefore recommended over linear regression models constructed for each individual. Linear mixed-effects models can account for both fixed effects at the population level and random effects, which allow for subject-specific intercepts and slopes—e.g. individual-specific variation from the population mean. We introduce the practical implications of linear mixed-effects models using R programming: calculating an individual’s eGFR slope and comparing mean eGFR slopes between groups. This article is based on the hands-on seminar at the annual meeting of the 68th Japanese Society of Nephrology in 2025.

**Supplementary Information:**

The online version contains supplementary material available at 10.1007/s10157-026-02835-8.

## Introduction

Kidney function, as measured by estimated glomerular filtration rate (eGFR), declines throughout the human lifespan due to physiological changes associated with aging [1–6]. In patients with kidney diseases, such as diabetic kidney disease [7, 8] and IgA nephropathy [9], the decline in eGFR is steeper than in healthy individuals. A steeper eGFR decline is associated with a higher incidence of kidney failure [10–12] or cardiovascular outcomes [13]. Recently, eGFR slope—the annual eGFR change—has been increasingly used as a surrogate endpoint for progression of chronic kidney disease (CKD), based on meta-analyses that showed a strong association between treatment effects on clinical endpoints (e.g., hard endpoints such as end-stage kidney disease or doubling of serum creatinine) and treatment effects on eGFR slope [14]. Regulatory authorities such as the US Food and Drug Administration (FDA) and the European Medicines Agency (EMA) have suggested that eGFR slope can be used as a surrogate endpoint for CKD progression [15]. Accordingly, there is an increasing number of observational studies that calculate the eGFR slope as an outcome [16–18] or exposure of interest [10–13] as well as randomized controlled trials.

To estimate an eGFR slope, repeated measurements of eGFR are taken from the same individual over time. When calculating the eGFR slope for each individual, a common approach has been to use a linear mixed-effects model that accounts for both fixed and random effects with subject-specific intercepts and slopes. As shown later, the linear mixed-effects model approach is considered statistically more efficient than linear regression models constructed for each individual. While advanced techniques, such as a two-slope model using a linear spline model with a linear mixed-effects model, are emerging [19], this paper focuses on understanding and implementing linear mixed-effects models as the fundamental approaches to calculate the eGFR slope in clinical research. By elucidating the principles and practical implementation of linear mixed-effects models, this article aims to facilitate readers’ basic comprehension and provide a steppingstone for advancing to more sophisticated analytical methods in subsequent research. Readers interested in the theoretical background of linear mixed-effects models are referred to the textbooks [20, 21]. This article is based on the hands-on seminar at the annual meeting of the 68th Japanese Society of Nephrology in 2025 [22].

## How to calculate individuals’ eGFR slope by a linear mixed-effects model

In a linear mixed-effects model used to calculate eGFR slope from repeated measurements on individuals, eGFR is expressed as a linear function of time.

$${\mathrm{eGFR}}_{ij}={\beta }_{0}+{\beta }_{1}\times {\mathrm{time}}_{ij}+{b}_{0i}+{b}_{1i}\times {\mathrm{time}}_{ij}+{\epsilon }_{ij}$$

Indices (*i* for individuals and *j* for time points, as shown below) are used to specify which measurement belongs to which person at which time point:

- The subscript $i$ represents the individual patient. This acknowledges that the data contains multiple measurements collected from the same person over time.
- The subscript $j$ represents the measurement time point for each patient $i$. A term like ${\mathrm{eGFR}}_{ij}$ refers to the eGFR value measured at time point $j$ for individual $i$.
- ${\mathrm{time}}_{ij}$ indicates the time variable (e.g., year) when the eGFR is measured at time point $j$ for individual $i$.
- ${\epsilon }_{ij}$ is a measurement error at time point $j$ for individual $i$ and is assumed to follow a normal distribution with a mean of 0 and a variance of ${\upsigma }^{2}$.

This model includes both fixed effects and random effects:

- Fixed effects (${\beta }_{0}$,${\beta }_{1}$) represent the population parameters $.$ ${\beta }_{0}$ is the fixed effect for the intercept (the average intercept for the population).${\beta }_{1}$ is the fixed effect for time (the average eGFR slope per year for the population).
- Random Effects ${b}_{0i}$ and ${b}_{1i}$ allow individual-specific deviations for the intercept and slope, respectively.

To apply the linear mixed-effects model with statistical software R, we use the hypothetical example dataset provided in the supplemental file. The dataset contains five variables: subject_id, group, time, egfr, and base_egfr. Note that the “group” is not used in this section but is used in the later section (“How to Compare mean eGFR slopes between two groups by a linear mixed-effects model”).

- **subject_id**: Identifier number for each participant
- **group**: Group to which the participant belongs (A or B)
- **time**: Time points of measurement (0, 1, 2, 3). The time is measured in years.
- **egfr**: eGFR value (mL/min/1.73 m^2^) at each time point
- **base_egfr**: eGFR value (mL/min/1.73 m^2^) at the baseline

The entire dataset (demo.csv in a supplemental file) contains 200 pseudo-individuals (subject_id) observations, with up to 4 measurement time points (time) per individual (baseline and 3 subsequent time points). For example, the first row indicates that subject 1 belonging to B had an eGFR value of 77.60 mL/min/1.73 m^2^ at time 0 and an eGFR value of 63.98 mL/min/1.73 m^2^ at time 1 (Table 1).

**Table 1:** Data structure

subject_id	Group	Time	egfr	base_egfr
1	B	0	77.60	77.60
1	B	1	63.98	77.60
1	B	2	70.99	77.60
1	B	3	64.86	77.60
2	B	0	85.56	85.56
2	B	1	74.69	85.56
2	B	2	66.12	85.56
2	B	3	73.99	85.56
3	B	0	76.07	76.07
3	B	1	71.75	76.07


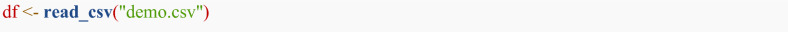


R code for a linear mixed-effects model using nlme package [23] is:
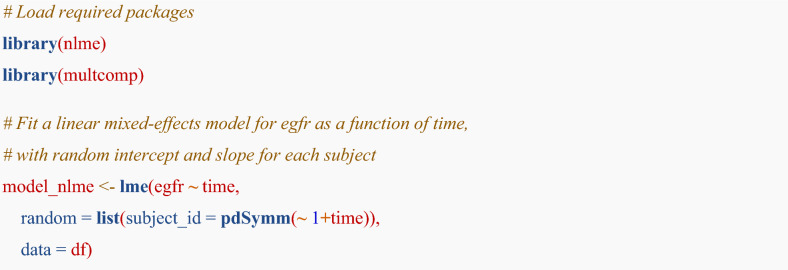


Note: In the code blocks, lines beginning with # represent comments, while lines starting with ## denote example output.

The details of the model specification are explained below:

- Fixed effects: **egfr ~ time**
- This estimates the population-level linear relationship between time and eGFR using a regression of eGFR on time. It assumes a common intercept and slope (time coefficient) for all subjects.
- Random effects: **list(subject_id = pdSymm(~ 1 + time))**

This allows for individual-level variation in two elements:

- Random intercepts: Variability in baseline eGFR between subjects.
  - Random slopes: Variability in the time effect (eGFR slope) for each subject.
  - **pdSymm()** specifies an unstructured covariance matrix for random effects ${b}_{0i}$ and ${b}_{1i}$ that:
    $$\mathbf{G}=\left[\begin{array}{cc}{\sigma }_{0}^{2}& {\sigma }_{01}\\ {\sigma }_{01}& {\sigma }_{1}^{2}\end{array}\right]$$

This matrix includes the following three parameters:

1. Intercept variance of random effect $\left({\sigma }_{0}^{2}\right)$
2. Slope variance of random effect $\left({\sigma }_{1}^{2}\right)$
3. Covariance between intercept and slope of random effect $\left({\sigma }_{01}\right)$

Extracting fixed Effects (fixef)

Once the model (model_nlme) is fitted, you can extract the fixed effects using the **fixef()** function.
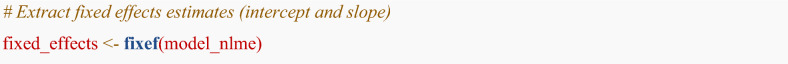


Extracting random effects (ranef)

We can extract the random effects using the **ranef()** function. These effects represent the deviation of each individual’s intercept and slope from the population averages.
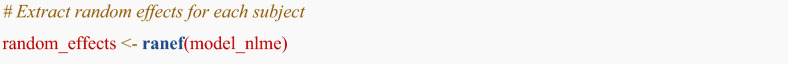


The eGFR slope for an individual *i* is calculated by adding the population average fixed slope (${\beta }_{1}$) and the individual random slope (${b}_{1i}$). Similarly, the individual intercept is the sum of the fixed intercept (${\beta }_{0}$) and the individual random intercept (${b}_{0i}$).

- **Individual eGFR slope**: ${\beta }_{1}+{b}_{1i}$
- **Individual eGFR intercept**: ${\beta }_{0}+{b}_{0i}$

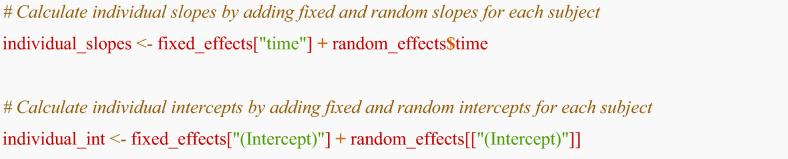


The calculated eGFR slopes for individuals were shown below. For example, − 2.66 mL/min/1.73 m^2^/year for subject_id 1, − 2.71 mL/min/1.73 m^2^/year for subject_id 2, and − 3.41 mL/min/1.73 m^2^/year for subject_id 3. Using the eGFR slope for individuals, researchers may subsequently conduct a descriptive study (e.g., summarizing the distribution of eGFR slopes overall) or an inference or prognostic study (e.g., factors associated with eGFR slope or rapid decline of eGFR, the association between eGFR slope and clinical events).

## Comparison of distributions in eGFR slope obtained from linear regression and linear mixed-effects modeling approaches

**Fig. 1:**
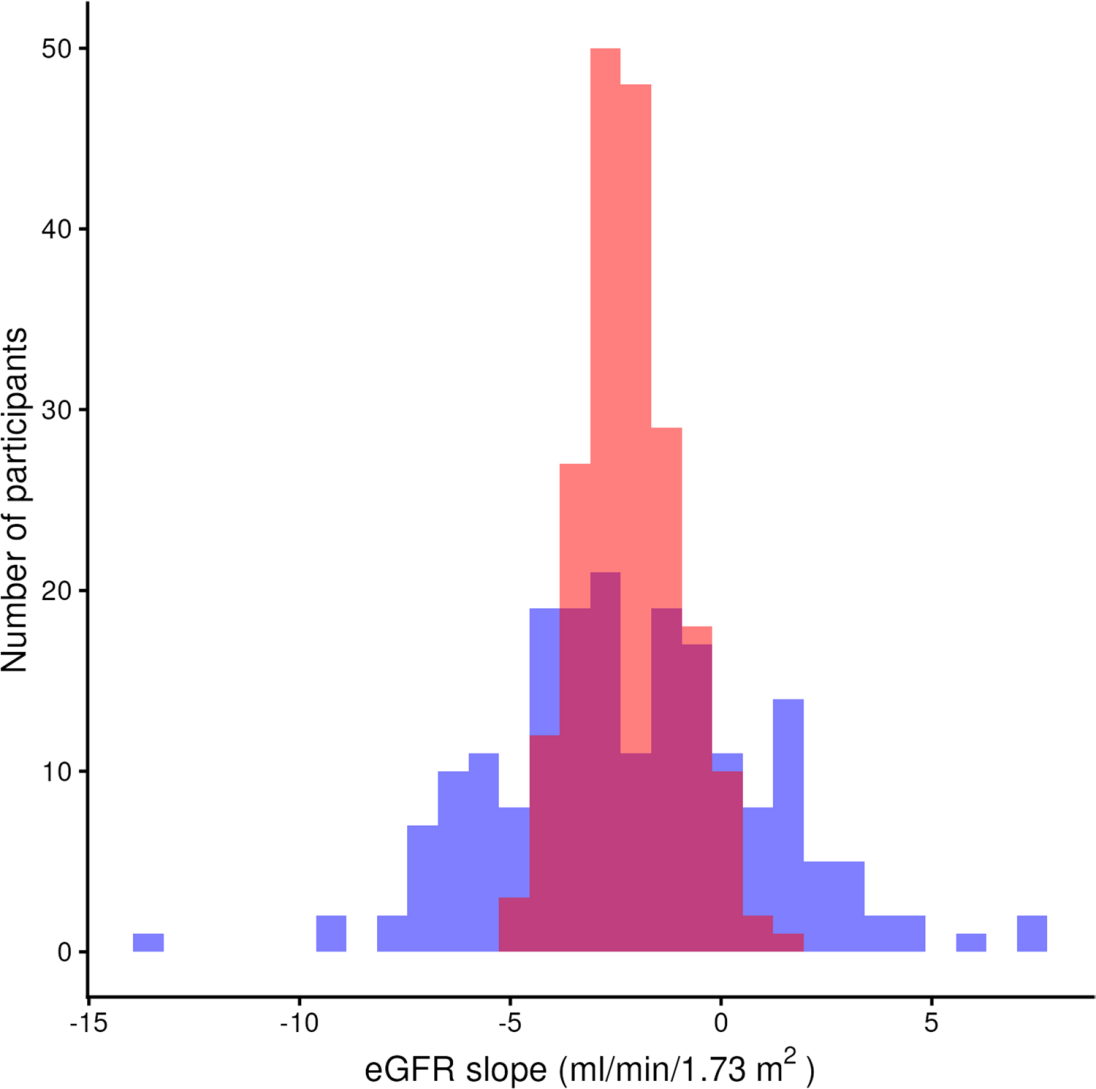
Comparison of the distributions of the eGFR slopes between the linear regression model and the linear mixed-effects model in the example dataset (N = 200). Blue indicates the distribution of eGFR calculated by fitting a linear regression model for each individual, and red indicates that by a linear mixed-effects model

The linear mixed-effects model approach is considered more efficient than the simple application of linear regression models for each individual (i.e., each individual’s eGFR slope is calculated by a linear regression model using only an individual’s eGFRs). Figure 1 shows distributions of eGFR slopes obtained from the linear regression and linear mixed-effects model approaches. In this hypothetical example, the variance of eGFR slope by linear regression is larger than that by linear mixed-effects model. An explanation of this phenomenon is presented below. Linear mixed-effects models assume that individual eGFR slopes are drawn from a common distribution of eGFR slope (e.g. all individuals). This assumption causes the estimates of each individual’s eGFR slope to shrink towards the overall mean slope of all individuals. This phenomenon, known as shrinkage, results in smaller standard errors of overall individual eGFR slopes by linear mixed-effects model compared to those estimated by linear regression in each individual. Shrinkage is most pronounced for individuals with few eGFR measurements, as the limited individual’s eGFR data is compensated by the model’s use of data from other individuals to improve estimation precision. The degree of shrinkage is dependent on the within-subject variance of eGFR measurements (${\upsigma }^{2}$) and between-subject variance of eGFR slopes (${\sigma }_{1}^{2})$. If ${\upsigma }^{2}\ll {\sigma }_{1}^{2}$, the individual slope estimated using linear mixed-effects model is close to that using simple linear regression in each individual. If ${\upsigma }^{2}\gg {\sigma }_{1}^{2}$, the estimate of ${b}_{1i}$ is close to zero. An individual’s eGFR slope derived from the linear mixed-effects model is also referred to as the “empirical Bayes” estimate. This is because the individual’s slope is derived from the estimated overall population mean (prior information) with the individual’s observations (likelihood). Thus, the individual’s eGFR slope can be interpreted as an estimate derived by borrowing from the population information, rather than relying solely on the individual's data.

In contrast, applying separate linear regression to each individual represents a markedly distinct methodology, where individual-specific parameters are estimated independently without assuming shared underlying distributions or borrowing information across individuals. This approach is particularly problematic when dealing with individuals who have only a few eGFR measurements, as the resulting slope estimates may be dominated by measurement error rather than true underlying physiological trends. In such cases, confidence intervals around individual slope estimates become substantially wider compared to linear mixed-effects models, reflecting the increased uncertainty inherent in individual-specific modeling without information borrowing.

In summary, the choice between these two approaches should depend on the specific purpose and context of use. Applying linear regression to each individual is a straightforward approach for individual-level clinical decision-making because it does not rely on population-level data. While this method is statistically inefficient [24] and highly sensitive to short-term variability and measurement errors, it may be appropriate for specific descriptive analyses focusing on individual trajectories when sufficiently dense and long follow-up data are available. On the other hand, slopes derived from linear mixed-effects are model-based estimates subject to shrinkage toward the population mean and should be interpreted as statistical estimates rather than directly observed trajectories. Due to their reliance on population data, linear mixed-effects models may be impractical for real-time clinical use. However, the linear mixed-effects model may be well suited for estimating population-level effects and group comparisons, particularly in datasets with heterogeneous follow-up.

## How to compare mean eGFR slopes between two groups by a linear mixed-effects model

Let us imagine a comparative effectiveness study evaluating the efficacy or effectiveness of a drug (drug A) over the other drug or placebo (drug B) in terms of eGFR slope. The difference in eGFR slope between groups A and B is interpreted as the efficacy or effectiveness of the target drug. For example, the FLOW trial [25] used total eGFR slope as a confirmatory secondary outcome. The ongoing FIND-CKD trial [26] uses eGFR slope over a specific period as a primary endpoint. Although there may be several approaches to compare eGFR slopes between two groups, including advanced approaches differentiating acute and chronic components in the context of RCTs or observational studies, here we introduce the basics of comparison of eGFR slope between two groups using linear mixed-effects models.

To evaluate differences in eGFR between groups, in addition to the formula in the previous section (“How to calculate an individual’s eGFR slope by a linear mixed-effects model”), we need to add a group term. Furthermore, inclusion of an interaction term between group and time is necessary to express that the rate at which eGFR changes as time progresses differs between groups. Thus, the formula is shown below:

$$\begin{aligned} {\mathrm{eGFR}}_{ij} & = \beta_{1} \times {\mathrm{time}}_{ij} + \beta_{2} \times \left( {{\mathrm{group}}\;{\mathrm{B}}\;{\mathrm{indicator}}} \right) + \beta_{3} \times \left( {{\mathrm{group}}\;{\mathrm{A}}\;{\mathrm{indicator}}} \right) \\ & \quad + \beta_{4 } \times {\mathrm{time}}_{ij} \times \left( {{\mathrm{group}}\;{\mathrm{A}}\;{\mathrm{indicator}}} \right) + b_{0i} + b_{1i} \times {\mathrm{time}}_{ij} + \varepsilon_{ij} \\ \end{aligned}$$

where the group A indicator is 1 if group = “A” and the group A indicator is 0 otherwise, and the group B indicator is 1 if group = “B” and the group B indicator is 0 otherwise. This equation represents a no-intercept parameterization. By removing the common intercept, we directly estimate group-specific intercepts (${\beta }_{2}$ and ${\beta }_{3}$).

- ${\beta }_{1}$ represents the rate in eGFR per 1-unit increase in time (e.g. one year in this example) in group B. A negative ${\beta }_{1}$ value indicates a decline in eGFR.
- ${\beta }_{2}$ and ${\beta }_{3}$ represent the eGFR intercept in groups B and A, respectively
- ${\beta }_{4}$ represents the difference in the rate of eGFR change over time between groups (i.e., the interaction effect between time and group)

**Fig. 2:**
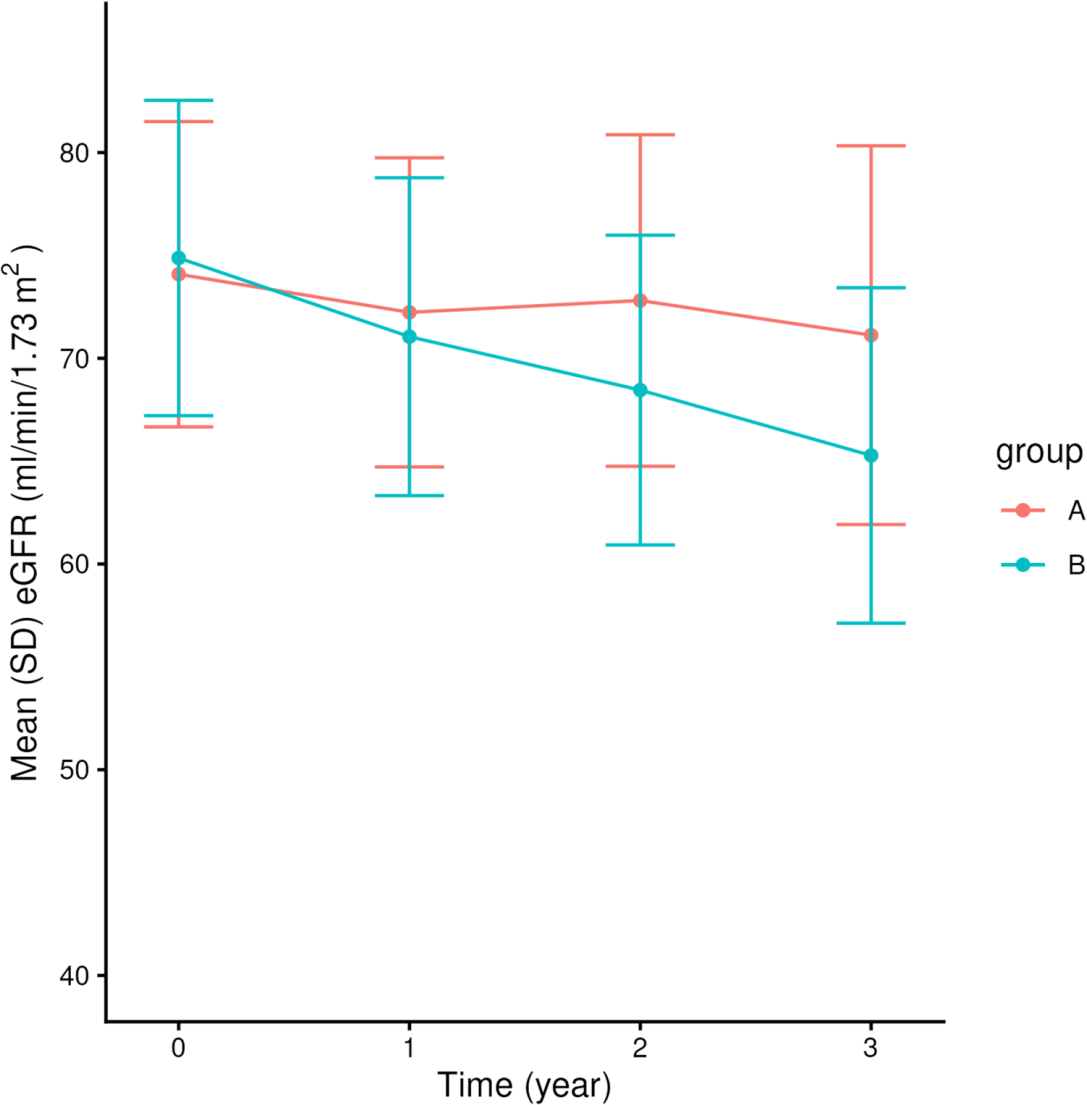
The trajectory of eGFR of the two groups in the example dataset (N = 200). The plot shows the mean eGFR with standard deviation at each timepoint by group

Figure 2 shows the mean eGFR values with standard deviations at each time point by groups. As shown, the difference in the mean eGFR between the groups increases incrementally over time.

To apply this formula to the example dataset of 200 pseudo-individuals, the R code is:
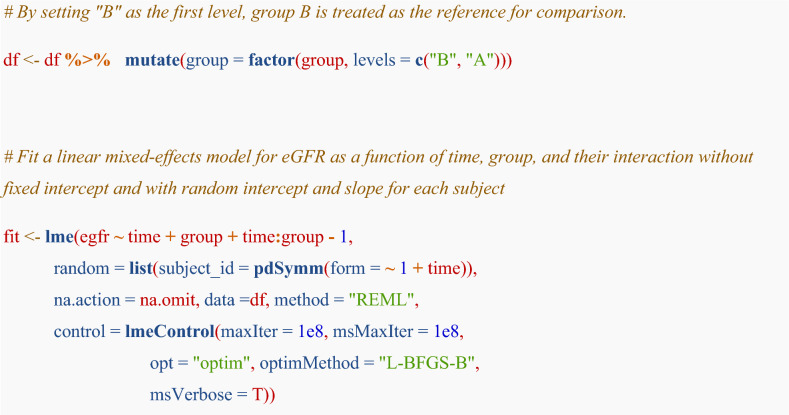


The details of the model specification are explained below:

**− 1**: Removes the intercept term of the fixed effect
- method = “REML”: Restricted Maximum Likelihood estimation
- maxiter = 1e8: Maximum optimization iterations
- opt = “optim”: Optimization algorithm
- optimMethod = “L-BFGS-B”: Box-constrained quasi-Newton method
- Note: The control arguments specified here (e.g., maxIter, msMaxIter) are included to ensure computational stability and the reproducibility of results. For this beginner’s guide, a detailed understanding of their internal mechanics is not required to apply the method effectively.

Due to the way R implements model specifications, the code specification here differs slightly from formula. Although we just specify the group variable in the code, R returns coefficients of group B and group A respectively after treating the group variable as factor.

Note that removing the intercept term from the fixed effect may not always be necessary; however, since the developers of the methodologies of the eGFR slope applied intercept suppression, [27, 28], and we followed their approach. We emphasize that omitting the fixed intercept is not a prerequisite for estimation of eGFR slopes. Instead, it is a parameterization strategy used to simplify interpretation (e.g., by directly estimating group-specific intercepts and slopes). We can further adjust for baseline eGFR as a covariate. Adjusting for baseline characteristics in the analysis of RCTs is advised by regulatory authorities such as the EMA and the US FDA because it may increase statistical efficiency [29]. In the observational study settings, to adjust for the baseline eGFR is warranted.

We can obtain point estimates of fixed effects with 95% confidence intervals using the R code:
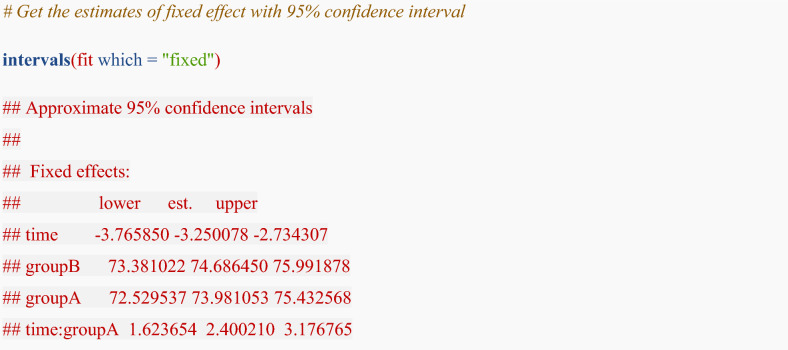


The time term in the fixed effects output corresponds to the (${\beta }_{1}$) coefficient. The estimated value and 95% confidence interval for this term indicate the magnitude of the eGFR slope in group B. The time:groupA term in the fixed effects output corresponds to the (${\beta }_{4}$) coefficient. The estimated value and 95% confidence interval for this term indicate the magnitude and significance of the difference in eGFR slope between group A and group B. The difference in eGFR slope between the groups was 2.40 [95% CI 1.62, 3.18] mL/min/1.73 m^2^ per year, meaning that the eGFR slope in group A was 2.40 mL/min/1.73 m^2^ per year less steep than that in group B.

To obtain the average eGFR slope for each group (group A and group B) and their 95% confidence intervals from the model results, we use contrast vectors (L) to combine the estimated fixed effects coefficients ($\beta$). The contrast simply instructs the software to combine the relevant fixed-effect coefficients (e.g., time and time × group). The following matrix notation is a formal representation of this otherwise straightforward operation.

For group A, the average slope is estimated by ${\beta }_{1}+{\beta }_{4}$ (the coefficient for time plus the coefficient for the time:groupA interaction). The contrast vector for this is **c(1, 0, 0, 1)**. To apply this calculation, the “multcomp” package is required:

To test the linear combination ${\beta }_{1}+{\beta }_{4}$, the following contrast vector is required:

$$L=\left[\begin{array}{cccc}1& 0& 0& 1\end{array}\right]$$

By setting L as above, L $\beta$ indicates the average eGFR slope for group A.

$$\left[\begin{array}{cccc}1& 0& 0& 1\end{array}\right]\times \left[\begin{array}{c}{\beta }_{1}\\ {\beta }_{2}\\ {\beta }_{3}\\ {\beta }_{4}\end{array}\right]=\left[\begin{array}{c}1\cdot {\beta }_{1}+0\cdot {\beta }_{2}+0\cdot {\beta }_{3}+1\cdot {\beta }_{4}\end{array}\right]=\left[\begin{array}{c}{\beta }_{1}+{\beta }_{4}\end{array}\right]$$

Note: In R, the coefficient names and their order should be confirmed from the model output (e.g., fixef(fit)) when constructing contrast vectors. Because R returns the coefficients (e.g. ${\beta }_{1}$ to ${\beta }_{4}$) in this order from the regression model, the contrast vectors must be constructed accordingly. Specifically, each element in the contrast vector L = [1, 0, 0, 1] corresponds to the fixed-effect coefficients in the order they appear in the model output: ${\beta }_{1}$ (time, e.g. the rate in eGFR per 1-unit increase in time in group B), ${\beta }_{2}$ (Group A intercept), ${\beta }_{3}$ (Group B intercept), and ${\beta }_{4}$ (time and Group A interaction, e.g. the difference in the rate of eGFR change over time between groups). By assigning a weight of 1 to ${\beta }_{1}$ and ${\beta }_{4}$ and a weight of 0 to the group-specific intercepts ${\beta }_{2}$ and ${\beta }_{3}$, we isolate the specific linear combination that defines the slope for Group A.

The R code is:
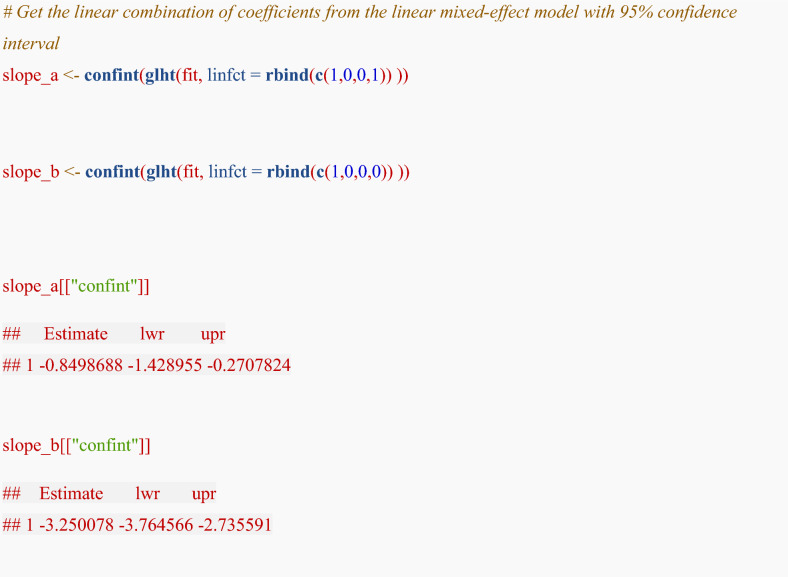


The eGFR slope for group A was − 0.85 [95% CI − 1.43, − 0.27] mL/min/1.73 m^2^ per year. The eGFR slope for group B was − 3.25 [95% CI − 3.76, − 2.74] mL/min/1.73 m^2^ per year.

## How long and how many measurements are needed to consider eGFR slope?

There are some controversies about the optimal duration to estimate eGFR slope. The EMA recommends a minimum follow-up period of two years, with three years preferred, in clinical trials evaluating CKD therapies in 2023 [30]. In the IMAGINE trial (NCT03744910), which evaluated the effect of clazakizumab for chronic active antibody-mediated rejection, FDA accepted the slope of eGFR over 12 months as a reasonably likely surrogate endpoint [31]. The trial was terminated in 2024 due to futility at the interim analysis [32].

This extended duration aims to enhance the reliability of eGFR slope calculations by reducing the impact of short-term variability. However, these guidelines specifically apply to interventional studies rather than observational research. To our knowledge, no established consensus exists for observational studies, where duration selection depends on the study's design and objectives. While increasing measurement frequency and extending the intervals between measurements theoretically improves slope precision, no universal standard has been established for optimal testing intervals in observational studies. A recent study showed that short-term eGFR variability can significantly impact assessment of eGFR slope, particularly when follow-up duration is limited [33].

## Addendum: difference in standard error by different approximation methods in a linear mixed-effects model

Please note that the main analyses in this tutorial rely on large-sample (normal) approximation. This addendum is provided as an advanced cautionary example to illustrate how inference may change in small-sample settings. The intervals function in the nlme package uses a normal approximation to obtain approximate confidence intervals for the parameters of a linear mixed-effects model [23]. This method assumes that the restricted maximum likelihood estimator for the parameters follows a normal distribution. The method may be valid in a large dataset; however, the type 1 error rate may be inflated, especially in a small sample dataset. The Satterthwaite [34] or Kenward–Roger [35, 36] methods are generally recommended. While the nlme package does not cover these methods, the lmerTest package can apply these methods [37]. To illustrate the difference in small-sample analysis, we present results from 30 randomly sampled subjects. For the illustration, we will show normal approximation method by using nlme package and Satterthwaite or Kenward–Roger method by using lmerTest package.

For assessing the difference in eGFR slope between the groups, R codes are shown below:
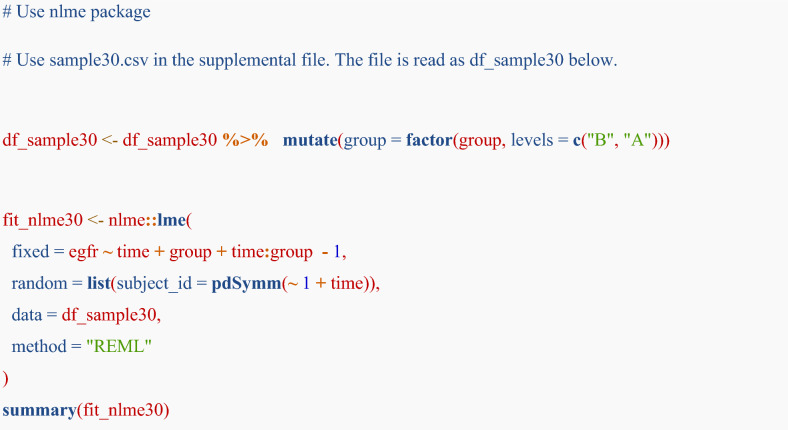



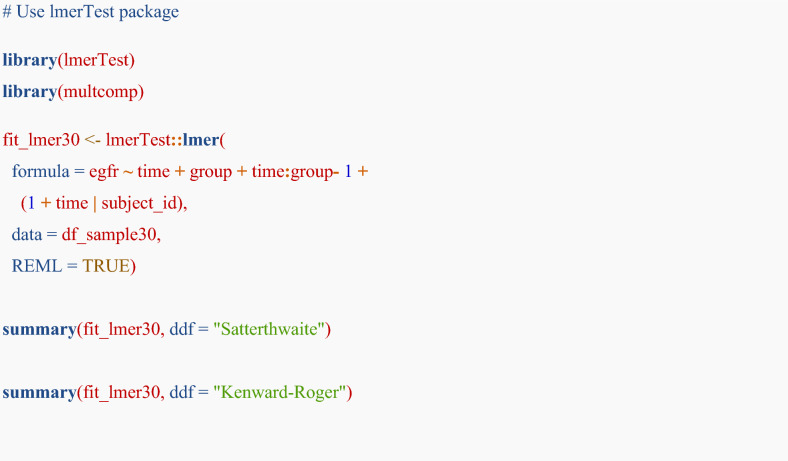


In the lme4 or lmerTest packages, specifying the random effects structure as (1 + time | subject_id) allows the model to estimate both the variances and the covariance of the random intercept and slope for each subject, which is equivalent to the pdSymm structure in the nlme package.

Table 2 indicates the differences in the outputs by method. The “Estimates” indicate the point estimates, which are almost similar by method. Std.Error and df indicates standard error and degree of freedom, respectively. Generally, Kenward–Roger method provides accurate but conservative results (e.g. larger standard error). Because degrees of freedom are adjusted in Satterthwaite and Kenward–Roger methods, those are lower than that of normal approximation method (nlme package).

**Table 2:** The difference in eGFR slope between the groups in the example dataset (N = 30), estimated by normal approximation, Satterthwaite and Kenward–Roger methods

Method	Estimate	Std. Error	df	t value	*P* value
Normal approximation	3.12714	1.26876	68.00000	2.46472	0.01624
Satterthwaite	3.12714	1.26876	24.11286	2.46473	0.02122
Kenward–Roger	3.12714	1.27497	26.33446	2.45273	0.02111

df, degree of freedom; Std. Error, standard error

To obtain 95% confidence interval of difference in eGFR slope between the groups, R codes are shown below:
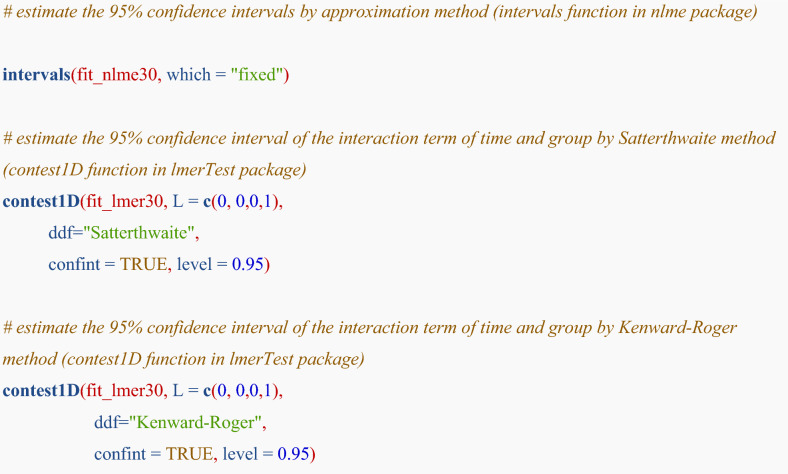


Table 3 indicates the point estimates with 95% confidence intervals by methods. As standard error of Kenward–Roger was largest, 95% confidence interval by Kenward–Roger was the widest among the methods.

**Table 3:** The difference in eGFR slope between the groups with 95% confidence interval in the example dataset (N = 30), estimated by normal approximation, Satterthwaite and Kenward–Roger methods

Method	Estimate	Lower	Upper
Normal approximation	3.12714	0.59537	5.65891
Satterthwaite	3.12714	0.50920	5.74508
Kenward–Roger	3.12714	0.50803	5.74625

The columns of lower and upper denote the lower and upper boundary of 95% confidence interval, respectively

## Conclusions

This paper provided a step-by-step guide for calculating individual eGFR slopes and comparing group differences using linear mixed-effects models in R. These analytical techniques may be essential for clinicians and researchers involved in kidney research, including both RCTs and observational studies.

## Supplementary Information

Below is the link to the electronic supplementary material.

Supplementary file1 (CSV 14 KB)

Supplementary file2 (CSV 2 KB)

## Data Availability

The artificially generated data used in this study are available as supplementary files. R code is also provided as a supplementary file.
